# Enrollment of Older Patients, Women, and Historically Underrepresented Racial and Ethnic Groups in Pulmonary Embolism Trials: A Systematic Review

**DOI:** 10.1016/j.jscai.2025.103993

**Published:** 2025-10-30

**Authors:** Sana Rehman, Tayyab Shah, Yousuf Shah, Andrew Schwartz, Kriyana P. Reddy, Lauren Glassmoyer, Taisei Kobayashi, Allison Tsao, Ashwin Nathan, Jay S. Giri, Alexander C. Fanaroff

**Affiliations:** a Department of Internal Medicine, Guy’s and St Thomas’ NHS Foundation Trust, London, United Kingdom; b Division of Cardiovascular Medicine, University of Pennsylvania, Philadelphia, Pennsylvania; c Department of Internal Medicine, University of Rochester, Rochester, New York; d Yale School of Medicine, New Haven, Connecticut

**Keywords:** age, enrollment, pulmonary embolism, race, women

## Introduction

The management of pulmonary embolism (PE) has been rapidly evolving over the past decade with new endovascular treatment strategies.^1^ Similar to other areas of cardiovascular medicine with recent advancements in invasive management,^2^^,^^3^ racial and sex disparities in management and outcomes of PE have become apparent.^4^^,^^5^ While addressing these disparities requires a multipronged approach, one of the first steps in any rapidly developing field is to evaluate the representativeness of clinical trials studying novel treatment strategies. Representative clinical trials are important for generalizability, ensuring equitable access to new technologies and increasing trust in the health care system and research findings.^4^^,^^6^^,^^7^ In this letter, we present the results of a systematic review addressing the representativeness of large, practice-informing PE prospective trials.

## Materials and methods

This review followed the Preferred Reporting Items for Systematic Reviews and Meta-Analyses (PRISMA) reporting guidelines, and the protocol was preregistered with PROSPERO (CRD420251029801). PubMed was searched on April 10, 2025, with search terms including pulmonary embolism/thromboembolism, treatment, prospective, trial, and registry. Results were limited to clinical trials and studies published in English on or after January 1, 2000. The bibliographies of potentially relevant publications and other reviews in the search were examined for additional relevant studies. Two independent reviewers among 4 reviewers (A.S., S.R., T.S., Y.S.) screened each title and abstract to identify potentially relevant studies, screened the remaining full texts, and abstracted data from relevant studies (with a third reviewer resolving disagreements). Inclusion criteria were any prospective study testing an intervention (eg, medical, endovascular, or surgical) on patients with acute PE, which enrolled ≥100 patients with at least 1 patient enrolled in North America (where the National Institutes of Health and the U.S. Food and Drug Administration mandate reporting of race and sex). Exclusion criteria were studies enrolling patients <18 years of age, studies reporting phase I or pilot studies, or secondary analyses of larger trials that enrolled patients other than those with acute PE (eg, deep vein thrombosis). The risk of bias for each study’s results was not performed because this systematic review does not address the outcomes of these studies.

We present general characteristics and enrollment demographic characteristics (age, sex, and race) of included trials. Continuous variables are presented as mean (SD) or as median (IQR). Categorical variables are presented as numbers and percentages. All calculated means were weighted by trial sample size. To characterize trends over time, we estimated linear regression models (weighted by trial sample size) using an enrollment demographic characteristic as the dependent variable (mean age, proportion of women, and proportion of non-White participants) and date of last patient enrollment as the independent variable. To quantify the representation of women, Black individuals, and Hispanics in PE trials, we calculated participation-to-prevalence ratios (PPRs). The PPR is defined as the percentage of individuals from a given demographic characteristic in a clinical trial population divided by the percentage of individuals from that demographic characteristic among all people with the disease. We calculated PPRs separately for women, Black race, and Hispanic ethnicity, using a large, nationally representative cohort derived from the National Inpatient Sample to determine the sex, racial, and ethnic composition of the population with PE admitted to US hospitals.^5^ In this study, mean age was 62 years; 52.2% of patients were female sex, 20.5% were Black, and 5.5% were Hispanic. By convention, PPRs between 0.8 and 1.2 indicate similar representation in the trial population and the overall disease population.^7^ Analyses were performed with R Studio version 4.4.1 (R Core Team).

## Results

**Figure 1:**
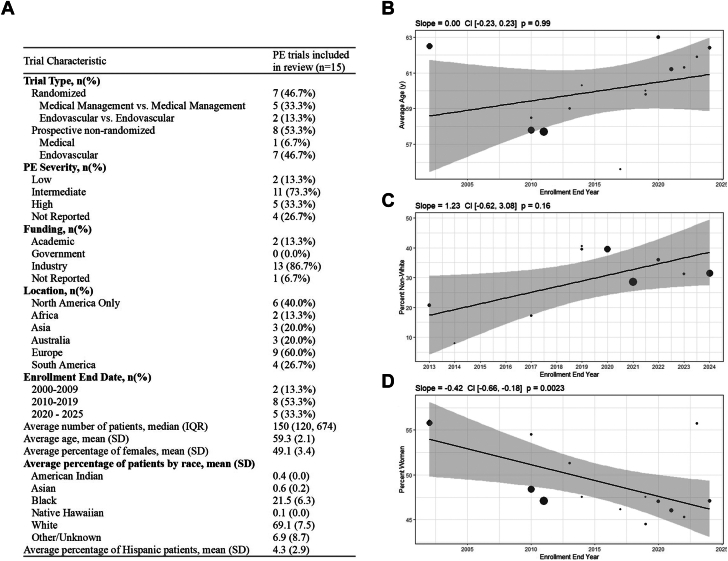
**Characteristics and demographics of the patient population in pulmonary embolism clinical trials.** (**A**) Pulmonary embolism (PE) study characteristics of included studies, which included APEX-AV, Büller 2012, EINSTEIN-PE, EXTRACT-PE, FLARE, FLASH, Hull 2000, KNOCOUT PE, MATISSE Study, MOPETT, OPTALYSE PE, PEERLESS, PERFECT, SEATTLE II, and STRIKE-PE. The graphs report the time trends in enrollment by (**B**) mean age; (**C**) female sex; and (**D**) non-White participants.

The search yielded 1355 references with 15 studies (13,253 patients) ultimately meeting eligibility criteria (Supplemental Table S1). Briefly, 7 studies were randomized controlled trials, the median number of enrolled patients was 150, most studies enrolled patients with intermediate-risk and/or high-risk PE, and 86.7% of studies were industry funded (Figure 1). Of the 15 trials, 93% reported average age, 100% reported female sex, 67% reported race, and 53% reported ethnicity. The mean age of patients across studies was 59.3 years, 49.1% were women, 21.5% were Blacks, and 4.3% were Hispanics. PPRs were 0.94 for female sex, 1.04 for Black race, and 0.78 for Hispanic ethnicity. There were no significant changes over time in mean age or enrollment of non-White patients; however, there was a significant decrease in proportion of enrolled females since 2000 (slope, −0.42%/y; 95% CI, −0.66 to −0.18; *P* = .002) (Figure 1). Among trials conducted exclusively in North America (n = 6), all reported age and sex, while only 67% and 33% reported race and ethnicity, respectively. PPRs for female sex, Black race, and Hispanic ethnicity were 1.00, 1.08, and 0.93, respectively.

## Discussion and conclusions

This study demonstrated that large, practice-informing PE clinical trials/prospective registries have enrolled a representative population in terms of age and sex. Race and ethnicity have not been consistently reported, but trials reporting race and ethnicity have enrolled a representative population, especially trials enrolling North American patients for whom the PPR calculation is most valid since population prevalence was measured in this population. Although there is limited evidence in this field with relatively few large, practice-informing trials, these results are important for 2 key reasons: first, they demonstrate that the existing evidence for the management of PE may be broadly generalizable. Second, they demonstrate that it is possible to enroll historically marginalized communities into clinical trials even among acutely ill patients, which has proven difficult in other fields. It is concerning, however, to see that the enrollment of female patients has steadily been falling for the past 20 years in PE clinical trials. The most recent trials have generally tested endovascular interventions requiring large-bore venous access. Women have smaller peripheral blood vessels and a higher risk of vascular complications with large-bore access, and this may have led trialists to approach fewer women for enrollment. However, the issue is likely multifactorial, and as endovascular strategies for PE management continue to develop, it will be critical for trialists to ensure appropriate enrollment of women, who make up about half of the disease population.

This systematic review has several limitations. First, it only included trials that were partly done in North America and may not reflect enrollment in non-North American trials. Second, not all trials reported race and ethnicity, and those trials reporting race and ethnicity may have differed in representativeness from trials that did. This limits our ability to definitively comment on the representativeness of PE trials with respect to race and ethnicity. Still, this study represents the most comprehensive study on this topic to date and indicates that, in contrast to other areas of cardiovascular medicine, clinical trials of management strategies for PE have enrolled representative populations.
