# Supplementary material for: Enrollment of Older Patients, Women, and Historically Underrepresented Racial and Ethnic Groups in Pulmonary Embolism Trials: A Systematic Review
Source: J Soc Cardiovasc Angiogr Interv. 2025 Oct 30;5(3 Suppl):103993. doi: 10.1016/j.jscai.2025.103993 (PMC13112788; doi:10.1016/j.jscai.2025.103993)
Supplement: Supplemental material [file mmc1.docx]

| Trial | Title | Trial type | Intervention | Location | # of patients | Average Age (y) | # of Male patients |
| --- | --- | --- | --- | --- | --- | --- | --- |
| APEX-AV^1^ | Novel Mechanical Aspiration Thrombectomy in Patients With Acute Pulmonary Embolism: Results From the Prospective APEX-AV Trial | Prospective Non-randomized trial | Endovascular | North America | 122 | 61.9 | 54 |
| STRIKE-PE^2^ | Clinical, Functional, and Quality-of-Life Outcomes after Computer Assisted Vacuum Thrombectomy for Pulmonary Embolism: Interim Analysis of the STRIKE-PE Study | Prospective Non-randomized trial | Endovascular | Europe; South America | 150 | 61.3 | 82 |
| PEERLESS^3^ | Large-Bore Mechanical Thrombectomy Versus Catheter-Directed Thrombolysis in the Management of Intermediate-Risk Pulmonary Embolism: Primary Results of the PEERLESS Randomized Controlled Trial | Randomized | Endovascular | Europe | 550 | 62.4 | 291 |
| FLASH^4^ | Acute outcomes for the full US cohort of the FLASH mechanical thrombectomy registry in pulmonary embolism | Prospective Non-randomized trial | Endovascular | Europe | 799 | 61.2 | 431 |
| KNOCOUT PE^5^ | Prospective Multicenter International Registry of Ultrasound-Facilitated Catheter-Directed Thrombolysis in Intermediate-High and High-Risk Pulmonary Embolism (KNOCOUT PE) | Prospective Non-randomized trial | Medical; Endovascular | Europe; Asia | 489 | 63 | 259 |
| EXTRACT-PE^6^ | Indigo Aspiration System for Treatment of Pulmonary Embolism: ResultsÂ ofÂ theÂ EXTRACT-PEÂ Trial | Prospective Non-randomized trial | Endovascular | North America | 119 | 59.8 | 66 |
| PERFECT^7^ | Pulmonary Embolism Response to Fragmentation, Embolectomy, and Catheter Thrombolysis (PERFECT): Initial Results From a Prospective Multicenter Registry | Prospective Non-randomized trial | Medical; Endovascular | Europe | 101 | 60.3 | 53 |
| FLARE^8^ | A Prospective, Single-Arm, Multicenter Trial of Catheter-Directed Mechanical Thrombectomy for Intermediate-Risk Acute Pulmonary Embolism: The FLARE Study | Prospective Non-randomized trial | Endovascular | North America | 104 | 55.6 | 56 |
| OPTALYSE PE^9^ | A Randomized Trial of the Optimum Duration of Acoustic Pulse Thrombolysis Procedure in Acute Intermediate-Risk Pulmonary Embolism: The OPTALYSE PE Trial | Randomized | Medical; Endovascular | Europe | 101 | 60 | 53 |
| MOPETT^10^ | Moderate pulmonary embolism treated with thrombolysis (from the "MOPETT" Trial) | Randomized | Medical | North America | 121 | 58.5 | 55 |
| SEATTLE II^11^ | A Prospective, Single-Arm, Multicenter Trial of Ultrasound-Facilitated, Catheter-Directed, Low-Dose Fibrinolysis for Acute Massive and Submassive Pulmonary Embolism: The SEATTLE II Study | Prospective Non-randomized trial | Medical; Endovascular | North America | 150 | 59 | 73 |
| Hull 2000^12^ | Low-molecular-weight heparin vs heparin in the treatment of patients with pulmonary embolism. American-Canadian Thrombosis Study Group | Randomized | Medical | North America | 200 | Not reported | 88 |
| EINSTEIN-PE^13^ | Oral rivaroxaban for the treatment of symptomatic pulmonary embolism | Randomized | Medical | Europe; Asia; South America; Australia; Africa | 4832 | 57.7 | 2556 |
| Büller 2012^14^ | Enoxaparin followed by once-weekly idrabiotaparinux versus enoxaparin plus warfarin for patients with acute symptomatic pulmonary embolism: a randomised, double-blind, double-dummy, non-inferiority trial | Randomized | Medical | Europe; Asia; South America; Australia; Africa | 3202 | 57.8 | 1652 |
| Matisse Study^15^ | Subcutaneous fondaparinux versus intravenous unfractionated heparin in the initial treatment of pulmonary embolism | Randomized | Medical | Europe; South America; Australia | 2213 | 62.5 | 978 |

**Supplementary Table:** trial characteristics

**Supplemental References**

1. Ranade M, Foster MT 3rd, Brady PS, et al. Novel Mechanical Aspiration Thrombectomy in Patients With Acute Pulmonary Embolism: Results From the Prospective APEX-AV Trial. *Journal of the Society for Coronary Angiography and Interventions*.2024;4(1):102463.
2. Moriarty JM, Dohad SY, Schiro BJ, et al. Clinical, Functional, and Quality-of-Life Outcomes after Computer Assisted Vacuum Thrombectomy for Pulmonary Embolism: Interim Analysis of the STRIKE-PE Study. *The Journal of Vascular and Interventional Radiology*. 2024;35(8):1154-1165.e6.
3. Jaber WA, Gonsalves CF, Stortecky S, et al. Large-Bore Mechanical Thrombectomy Versus Catheter-Directed Thrombolysis in the Management of Intermediate-Risk Pulmonary Embolism: Primary Results of the PEERLESS Randomized Controlled Trial. *Circulation*. 2025;151(5):260-273.
4. Toma C, Jaber WA, Weinberg MD, et al. Acute outcomes for the full US cohort of the FLASH mechanical thrombectomy registry in pulmonary embolism. *EuroIntervention*. 2023;18(14):1201-1212.
5. Sterling KM, Goldhaber SZ, Sharp ASP, et al. Prospective Multicenter International Registry of Ultrasound-Facilitated Catheter-Directed Thrombolysis in Intermediate-High and High-Risk Pulmonary Embolism (KNOCOUT PE). *Circulation: Cardiovascular Interventions*. 2024;17(3):e013448.
6. Sista AK, Horowitz JM, Tapson VF, et al. Indigo Aspiration System for Treatment of Pulmonary Embolism: Results of the EXTRACT-PE Trial. *JACC: Cardiovascular Interventions*. 2021;14(3):319-329.
7. Kuo WT, Banerjee A, Kim PS, et al. Pulmonary Embolism Response to Fragmentation, Embolectomy, and Catheter Thrombolysis (PERFECT): Initial Results From a Prospective Multicenter Registry. *CHEST*. 2015;148(3):667-673.
8. Tu T, Toma C, Tapson VF, et al. A Prospective, Single-Arm, Multicenter Trial of Catheter-Directed Mechanical Thrombectomy for Intermediate-Risk Acute Pulmonary Embolism: The FLARE Study. *JACC: Cardiovascular Interventions*. 2019;12(9):859-869.
9. Tapson VF, Sterling K, Jones N, et al. A Randomized Trial of the Optimum Duration of Acoustic Pulse Thrombolysis Procedure in Acute Intermediate-Risk Pulmonary Embolism: The OPTALYSE PE Trial. *JACC: Cardiovascular Interventions*. 2018;11(14):1401-1410.
10. Sharifi M, Bay C, Skrocki L, Rahimi F, Mehdipour M; “MOPETT” Investigators. Moderate pulmonary embolism treated with thrombolysis (from the "MOPETT" Trial). *American Journal of Cardiology*. 2013;111(2):273-277.
11. Piazza G, Hohlfelder B, Jaff MR, et al. A Prospective, Single-Arm, Multicenter Trial of Ultrasound-Facilitated, Catheter-Directed, Low-Dose Fibrinolysis for Acute Massive and Submassive Pulmonary Embolism: The SEATTLE II Study. *JACC: Cardiovascular Interventions*. 2015;8(10):1382-1392.
12. Hull RD, Raskob GE, Brant RF, et al. Low-molecular-weight heparin vs heparin in the treatment of patients with pulmonary embolism. American-Canadian Thrombosis Study Group. *Archives of Internal Medicine*. 2000;160(2):229-236.
13. EINSTEIN–PE Investigators, Büller HR, Prins MH, et al. Oral rivaroxaban for the treatment of symptomatic pulmonary embolism. *New England Journal of Medicine*. 2012;366(14):1287-1297.
14. Büller HR, Gallus AS, Pillion G, Prins MH, Raskob GE; Cassiopea Investigators. Enoxaparin followed by once-weekly idrabiotaparinux versus enoxaparin plus warfarin for patients with acute symptomatic pulmonary embolism: a randomised, double-blind, double-dummy, non-inferiority trial. Lancet. 2012;379(9811):123-129.
15. Büller HR, Davidson BL, Decousus H, et al. Subcutaneous fondaparinux versus intravenous unfractionated heparin in the initial treatment of pulmonary embolism. *New England Journal of Medicine.* 2003;349(18):1695-1702.
